# Supplementary material for: Nearshore fish community changes along the Toronto waterfront in accordance with management and restoration goals: Insights from two decades of monitoring
Source: PLoS One. 2024 Feb 26;19(2):e0298333. doi: 10.1371/journal.pone.0298333 (PMC10896508; doi:10.1371/journal.pone.0298333)
Supplement: S1 Table — Kruskal-Wallis test and conover comparison (BH adjusted p-value) for years blocked into three periods (03–08 T1, 09–14 T2, 15–21 T3). (DOCX) [file pone.0298333.s001.docx]

Table S1.

| CPUE |  |  |  |  |
| --- | --- | --- | --- | --- |
| Ecotype | Species | Chi-squared | df | p-value |
| Open coast | All | 4.5902 | 2 | 0.1 |
| Pairwise conover |  |  | Time Period |  |
|  |  |  | T1-T2 | 0.0971 |
|  |  |  | T1-T3 | 0.2682 |
|  |  |  | T2-T3 | 0.0539 |
| Open coast | Alewife, Emerald | 5.8256 | 2 | 0.04* |
| Pairwise conover |  |  | Time Period |  |
|  |  |  | T1-T2 | 0.0726 |
|  |  |  | T1-T3 | 0.1890 |
|  |  |  | T2-T3 | 0.0219* |
| Open coast | Rest | 3.9464 | 2 | 0.14 |
| Pairwise conover |  |  | Time Period |  |
|  |  |  | T1-T2 | 0.1918 |
|  |  |  | T1-T3 | 0.1922 |
|  |  |  | T2-T3 | 0.0753 |
| Embayment | All | 7.2177 | 2 | 0.03* |
| Pairwise conover |  |  | Time Period |  |
|  |  |  | T1-T2 | 0.3448 |
|  |  |  | T1-T3 | 0.0131* |
|  |  |  | T2-T3 | 0.0156* |
| Embayment | Alewife, Emerald | 6.6511 | 2 | 0.04* |
| Pairwise conover |  |  | Time Period |  |
|  |  |  | T1-T2 | 0.4880 |
|  |  |  | T1-T3 | 0.0145* |
|  |  |  | T2-T3 | 0.0271* |
| Embayment | Rest | 3.0803 | 2 | 0.21 |
| Pairwise conover |  |  | Time Period |  |
|  |  |  | T1-T2 | 0.2744 |
|  |  |  | T1-T3 | 0.1409 |
|  |  |  | T2-T3 | 0.2018 |
| Coastal Wetland | All | 7.409 | 2 | 0.02* |
| Pairwise conover |  |  | Time Period |  |
|  |  |  | T1-T2 | 0.3551 |
|  |  |  | T1-T3 | 0.0132* |
|  |  |  | T2-T3 | 0.0118* |
| Coastal Wetland | Alewife, Emerald | 6.334 | 2 | 0.04* |
| Pairwise conover |  |  | Time Period |  |
|  |  |  | T1-T2 | 0.1463 |
|  |  |  | T1-T3 | 0.0619 |
|  |  |  | T2-T3 | 0.0149* |
| Coastal Wetland | Rest | 2.1248 | 2 | 0.35 |
| Pairwise conover |  |  | Time Period |  |
|  |  |  | T1-T2 | 0.3608 |
|  |  |  | T1-T3 | 0.2427 |
|  |  |  | T2-T3 | 0.2444 |
