# Supplementary material for: Nearshore fish community changes along the Toronto waterfront in accordance with management and restoration goals: Insights from two decades of monitoring
Source: PLoS One. 2024 Feb 26;19(2):e0298333. doi: 10.1371/journal.pone.0298333 (PMC10896508; doi:10.1371/journal.pone.0298333)
Supplement: S2 Table — Kruskal-Wallis test and conover comparison (BH adjusted p-value) for years blocked into three periods (03–08 T1, 09–14 T2, 15–21 T3). (DOCX) [file pone.0298333.s002.docx]

Table S2.

| BPUE |  |  |  |  |
| --- | --- | --- | --- | --- |
| Ecotype | Species | Chi-squared | df | p-value |
| Open coast | All | 9.4233 | 2 | 0.01* |
| Pairwise conover |  |  | Time Period |  |
|  |  |  | T1-T2 | 0.0495* |
|  |  |  | T1-T3 | 0.0241* |
|  |  |  | T2-T3 | 0.0011* |
| Open coast | C. Carp | 12.9444 | 2 | 0.002* |
| Pairwise conover |  |  | Time Period |  |
|  |  |  | T1-T2 | 0.0218* |
|  |  |  | T1-T3 | 0.00001* |
|  |  |  | T2-T3 | 0.0008* |
| Open coast | Rest | 3.7391 | 2 | 0.15 |
| Pairwise conover |  |  | Time Period |  |
|  |  |  | T1-T2 | 0.2030 |
|  |  |  | T1-T3 | 0.2007 |
|  |  |  | T2-T3 | 0.0863 |
| Embayment | All | 7.197 | 2 | 0.03* |
| Pairwise conover |  |  | Time Period |  |
|  |  |  | T1-T2 | 0.0596 |
|  |  |  | T1-T3 | 0.0074* |
|  |  |  | T2-T3 | 0.1036 |
| Embayment | C. Carp, W. Sucker | 10.9301 | 2 | 0.004* |
| Pairwise conover |  |  | Time Period |  |
|  |  |  | T1-T2 | 0.3523 |
|  |  |  | T1-T3 | 0.0006* |
|  |  |  | T2-T3 | 0.0007* |
| Embayment | Rest | 2.2301 | 2 | 0.33 |
| Pairwise conover |  |  | Time Period |  |
|  |  |  | T1-T2 | 0.1746 |
|  |  |  | T1-T3 | 0.2839 |
|  |  |  | T2-T3 | 0.4669 |
| Coastal Wetland | All | 4.8391 | 2 | 0.09 |
| Pairwise conover |  |  | Time Period |  |
|  |  |  | T1-T2 | 0.4557 |
|  |  |  | T1-T3 | 0.0736 |
|  |  |  | T2-T3 | 0.0460* |
| Coastal Wetland | C. Carp, F. Drum | 6.5992 | 2 | 0.04* |
| Pairwise conover |  |  | Time Period |  |
|  |  |  | T1-T2 | 0.1878 |
|  |  |  | T1-T3 | 0.0140* |
|  |  |  | T2-T3 | 0.0463* |
| Coastal Wetland | Rest | 4.4932 | 2 | 0.11 |
| Pairwise conover |  |  | Time Period |  |
|  |  |  | T1-T2 | 0.2562 |
|  |  |  | T1-T3 | 0.0601 |
|  |  |  | T2-T3 | 0.1074 |
