# Supplementary material for: Nearshore fish community changes along the Toronto waterfront in accordance with management and restoration goals: Insights from two decades of monitoring
Source: PLoS One. 2024 Feb 26;19(2):e0298333. doi: 10.1371/journal.pone.0298333 (PMC10896508; doi:10.1371/journal.pone.0298333)
Supplement: S3 Table — (DOCX) [file pone.0298333.s003.docx]

Table 3.

| RDA - CPUE | Permutations: | 999 |  |  |  |  |
| --- | --- | --- | --- | --- | --- | --- |
| Ecotype | df | SumsofSqs | MeanSqs | F-value | R^2^ | Pr(>F) |
| Open coast | 2 | 0.24071 | 0.120356 | 2.0874 | 0.20693 | 0.04* |
| Residuals | 16 | 0.92252 | 0.057657 |  | 0.79307 |  |
| T1-T2 | 1 | 0.06501 |  | 1.1611 | 0.10403 | 0.264 |
| Residuals | 10 | 0.55990 |  |  | 0.89597 |  |
| T1-T3 | 1 | 0.19031 |  | 2.7617 | 0.20068 | 0.013* |
| Residuals | 11 | 0.75801 |  |  | 0.79932 |  |
| T2-T3 | 1 | 0.10149 |  | 2.1179 | 0.16145 | 0.127 |
| Residuals | 11 | 0.52712 |  |  | 0.83855 |  |
| Ecotype | df | SumsofSqs | MeanSqs | F-value | R^2^ | Pr(>F) |
| Embayment | 2 | 0.57533 | 0.287665 | 4.1206 | 0.33997 | 0.01* |
| Residuals | 16 | 1.11697 | 0.069811 |  | 0.66003 |  |
| T1-T2 | 1 | 0.13811 |  | 1.9418 | 0.16261 | 0.134 |
| Residuals | 10 | 0.771124 |  |  | 0.83739 |  |
| T1-T3 | 1 | 0.25214 |  | 2.9092 | 0.20916 | 0.036* |
| Residuals | 11 | 0.95335 |  |  | 0.79084 |  |
| T2-T3 | 1 | 0.46124 |  | 8.9112 | 0.44755 | 0.003* |
| Residuals | 11 | 0.56936 |  |  | 0.55245 |  |
| Ecotype | df | SumsofSqs | MeanSqs | F-value | R^2^ | Pr(>F) |
| Coastal Wetland | 2 | 0.57063 | 0.28532 | 3.8745 | 0.32629 | 0.01* |
| Residuals | 16 | 1.17823 | 0.07364 |  | 0.67371 |  |
| T1-T2 | 1 | 0.15713 |  | 2.1651 | 0.17798 | 0.052 |
| Residuals | 10 | 0.72573 |  |  | 0.82202 |  |
| T1-T3 | 1 | 0.49668 |  | 6.0885 | 0.35629 | 0.002* |
| Residuals | 11 | 0.89734 |  |  | 0.64371 |  |
| T2-T3 | 1 | 0.19228 |  | 2.884 | 0.20772 | 0.014* |
| Residuals | 11 | 0.73340 |  |  | 0.79228 |  |
