# Supplementary material for: Nearshore fish community changes along the Toronto waterfront in accordance with management and restoration goals: Insights from two decades of monitoring
Source: PLoS One. 2024 Feb 26;19(2):e0298333. doi: 10.1371/journal.pone.0298333 (PMC10896508; doi:10.1371/journal.pone.0298333)
Supplement: S4 Table — (DOCX) [file pone.0298333.s004.docx]

Table 4.

| RDA - BPUE | Permutations: | 999 |  |  |  |  |
| --- | --- | --- | --- | --- | --- | --- |
| Ecotype | df | SumsofSqs | MeanSqs | F-value | R^2^ | Pr(>F) |
| Open coast | 2 | 0.19702 | 0.098508 | 1.4735 | 0.1554 | 0.16 |
| Residuals | 16 | 1.06966 | 0.66854 |  | 0.8446 |  |
| Ecotype | df | SumsofSqs | MeanSqs | F-value | R^2^ | Pr(>F) |
| Embayment | 2 | 0.34980 | 0.174898 | 4.2341 | 0.34609 | 0.01* |
| Residuals | 16 | 0.66091 | 0.041307 |  | 0.65391 |  |
| T1-T2 | 1 | 0.064144 |  | 1.5633 | 0.1352 | 0.198 |
| Residuals | 10 | 0.39302 |  |  | 0.8648 |  |
| T1-T3 | 1 | 0.21947 |  | 4.7957 | 0.30361 | 0.002* |
| Residuals | 11 | 0.50342 |  |  | 0.69639 |  |
| T2-T3 | 1 | 0.23505 |  | 6.0782 | 0.3559 | 0.008* |
| Residuals | 11 | 0.42538 |  |  | 0.6441 |  |
| Ecotype | df | SumsofSqs | MeanSqs | F-value | R^2^ | Pr(>F) |
| Coastal Wetland | 2 | 0.35433 | 0.177166 | 3.5593 | 0.30792 | 0.01* |
| Residuals | 16 | 0.79640 | 0.049775 |  | 0.69208 |  |
| T1-T2 | 1 | 0.14394 |  | 3.1433 | 0.23916 | 0.019* |
| Residuals | 10 | 0.45792 |  |  | 0.76084 |  |
| T1-T3 | 1 | 0.29656 |  | 5.4364 | 0.33076 | 0.001* |
| Residuals | 11 | 0.60005 |  |  | 0.66924 |  |
| T2-T3 | 1 | 0.08845 |  | 1.8191 | 0.14191 | 0.092 |
| Residuals | 11 | 0.53483 |  |  | 0.85809 |  |
