# Supplementary material for: Nearshore fish community changes along the Toronto waterfront in accordance with management and restoration goals: Insights from two decades of monitoring
Source: PLoS One. 2024 Feb 26;19(2):e0298333. doi: 10.1371/journal.pone.0298333 (PMC10896508; doi:10.1371/journal.pone.0298333)
Supplement: S5 Table — Kruskal-Wallis test and Dunn post-hoc (BH adjusted p-value) for years blocked into three periods (03–08 T1, 09–14 T2, 15–21 T3). (DOCX) [file pone.0298333.s005.docx]

Table 5.

| Biomass % | Ecotype | ɛ^2^ | CI 99% | p-value | Mean yearly Richness |
| --- | --- | --- | --- | --- | --- |
| Piscivores | Coastal wetland | 0.32 | 0.07, 1.00 | 0.04* | 0.03* |
|  |  |  | Time Period |  |  |
|  |  |  | T1-T2 | n.s. | n.s. |
|  |  |  | T1-T3 | 0.03* | n.s. |
|  |  |  | T2-T3 | n.s. | 0.026* |
|  | Embayment | 0.07 | 5.93e-04, 1.00 | 0.52 | n.s. |
|  | Open coast | 0.25 | 0.01, 1.00 | 0.05* | 0.0004* |
|  |  |  | Time Period |  |  |
|  |  |  | T1-T2 | n.s. | 0.0003 |
|  |  |  | T1-T3 | 0.04* | 0.0002 |
|  |  |  | T2-T3 | n.s. | n.s. |
| Specialists | Coastal wetland | 0.07 | 6.03e-04, 1.00 | 0.56 | n.s. |
|  | Embayment | 0.19 | 2.56e-03, 1.00 | 0.19 | n.s. |
|  | Open coast | 0.10 | 5.90e-04, 1.00 | 0.40 | n.s. |
