# Supplementary figures and images for: Nearshore fish community changes along the Toronto waterfront in accordance with management and restoration goals: Insights from two decades of monitoring
Source: PLoS One. 2024 Feb 26;19(2):e0298333. doi: 10.1371/journal.pone.0298333 (PMC10896508; doi:10.1371/journal.pone.0298333)

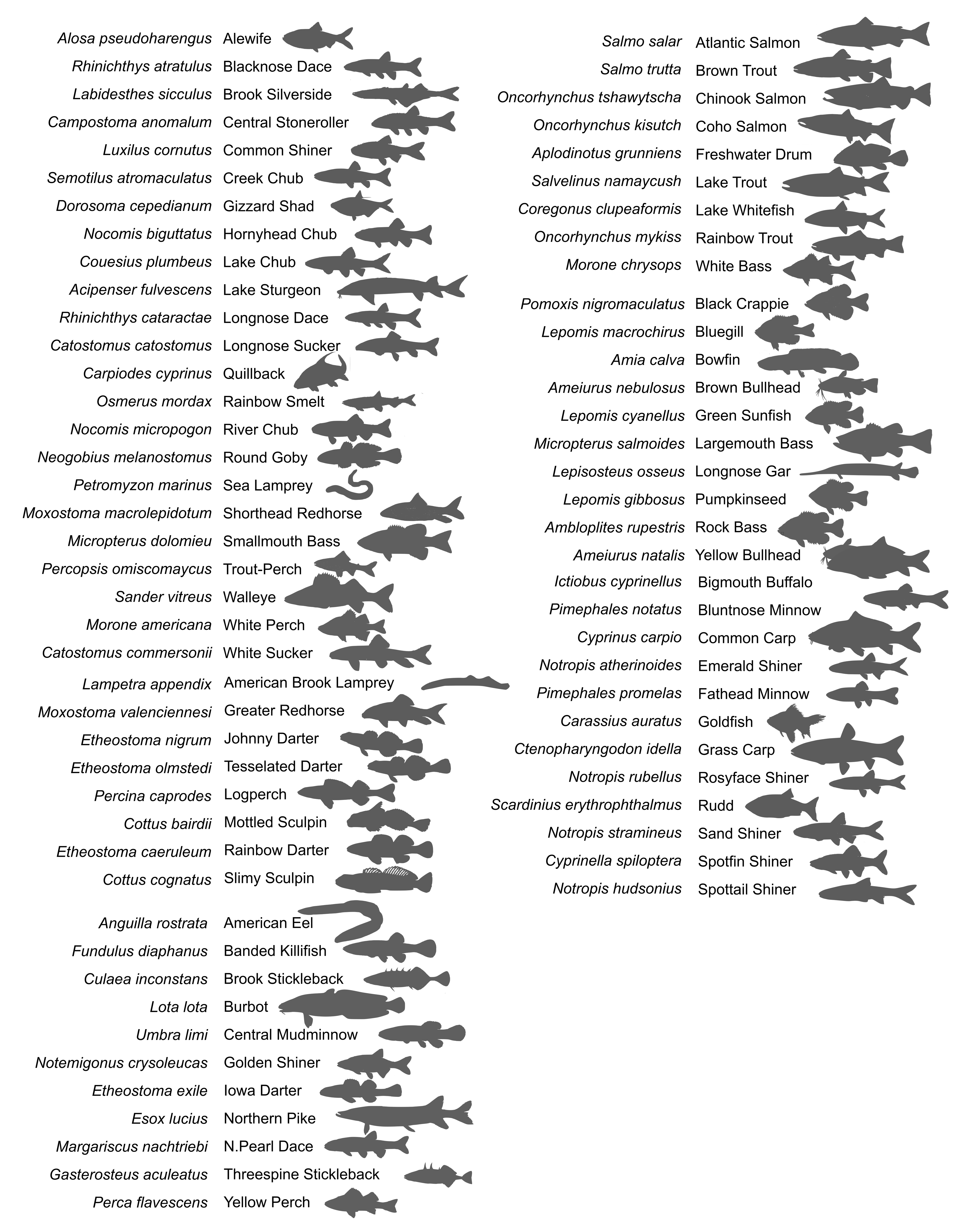

Supplement: S1 Fig — Waterfront fish species list and scientific names. (TIF) [file pone.0298333.s006.tif]

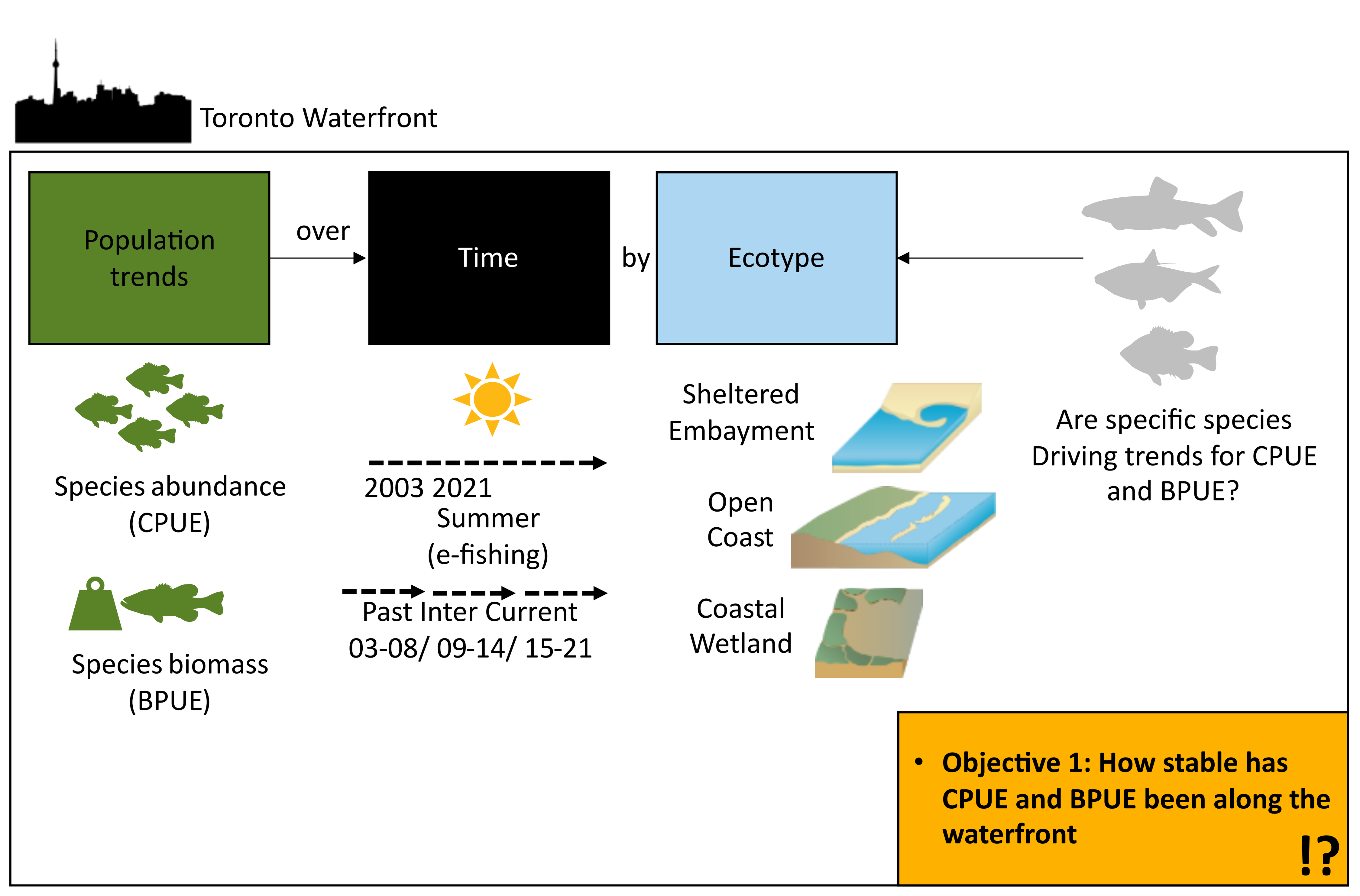

Supplement: S2 Fig — Workflow to analyze population trends for catch (CPUE) and biomass (BPUE) over time based on summer boat electrofishing data and across ecotypes (embayment, open coast, coastal wetland) between the years of 2003 and 2021. Years were blocked into three periods (03–08, 09–14, 15–21). Symbol attribution Tracey Saxby, Integration and Application Network; Kate Moore, Moreton Bay Waterways and Catchments Partnership (ian.umces.edu/media-library). Reprinted from ian.umces.edu/media-library under a CC BY 4.0 license, with permission from. (TIF) [file pone.0298333.s007.tif]

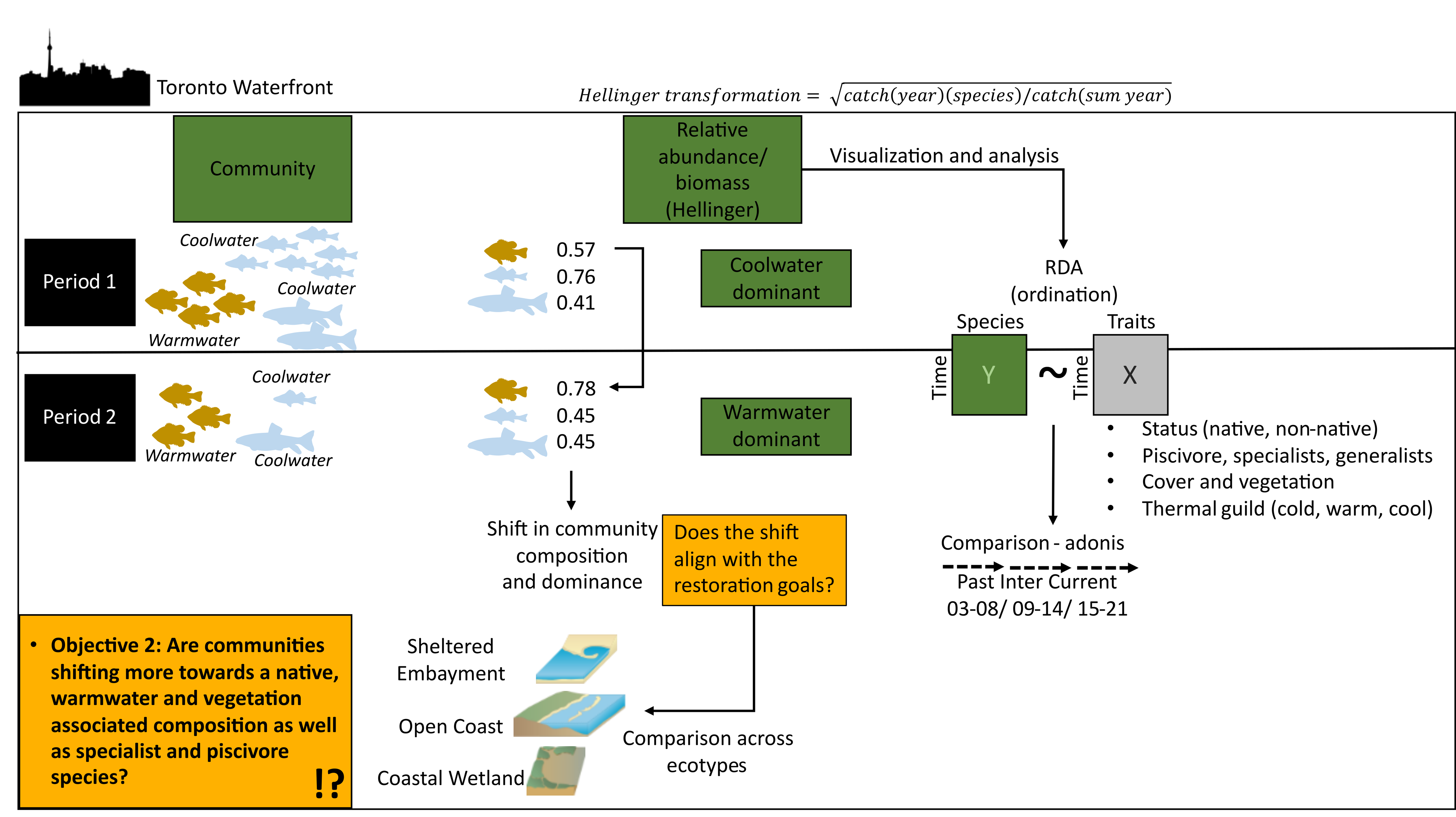

Supplement: S3 Fig — Workflow to analyze community changes based on relative catch and biomass (Hellinger transformed) over time based on summer boat electrofishing data and across ecotypes (embayment, open coast, coastal wetland) between the years of 2003 and 2021. Years were blocked into three periods (03–08, 09–14, 15–21). Included traits were status, feeding guild, habitat, and thermal guild and plotted in ordination space (RDA). Symbol attribution Tracey Saxby, Integration and Application Network; Kate Moore, Moreton Bay Waterways and Catchments Partnership (ian.umces.edu/media-library). Reprinted from ian.umces.edu/media-library under a CC BY 4.0 license, with permission from. (TIF) [file pone.0298333.s008.tif]

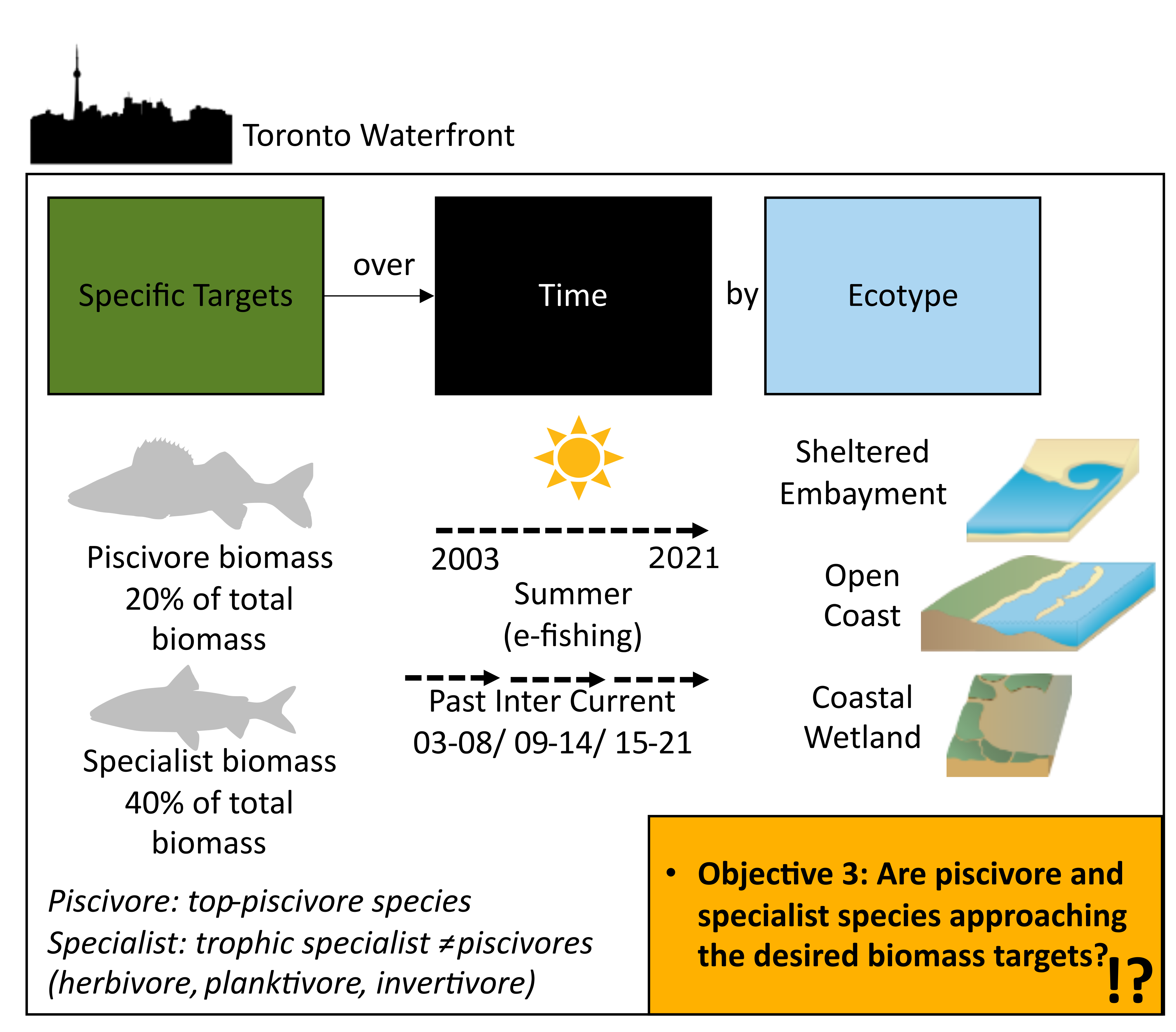

Supplement: S4 Fig — Workflow to analyze biomass contribution to total biomass for piscivore and specialist species over time based on summer boat electrofishing data and across ecotypes (embayment, open coast, coastal wetland) between the years of 2003 and 2021. Years were blocked into three periods (03–08, 09–14, 15–21). Symbol attribution Tracey Saxby, Integration and Application Network; Kate Moore, Moreton Bay Waterways and Catchments Partnership (ian.umces.edu/media-library). Reprinted from ian.umces.edu/media-library under a CC BY 4.0 license, with permission from ian.umces.edu/media-library, original copyright 2005 & 2010. (TIF) [file pone.0298333.s009.tif]
