# Supplementary material for: Nearshore fish community changes along the Toronto waterfront in accordance with management and restoration goals: Insights from two decades of monitoring
Source: PLoS One. 2024 Feb 26;19(2):e0298333. doi: 10.1371/journal.pone.0298333 (PMC10896508; doi:10.1371/journal.pone.0298333)
Supplement: Attachment — Submitted filename: Comments & Feedback.docx [file pone.0298333.s010.docx]

**Comments:**

1. Overall, the manuscript "Nearshore fish community changes along the Toronto Waterfront in accordance with management and restoration goals: Insights from two decades of monitoring" is a well-written and informative study.
2. The research aimed at broad spatiotemporal scale monitoring data from the Toronto Waterfront nearshore community to determine how the fish populations along the Toronto Waterfront have changed over the past two decades.
3. The authors use a long-term dataset of electrofishing data to examine changes in the nearshore fish community of the Toronto Waterfront. They identify a few key species that are driving changes in the community, and they discussed the potential implications of their findings for future management and restoration efforts.
4. The research data supports the conclusions and the experiments have been conducted with appropriate methodology and proper sample sizes based on appropriate data available.
5. The statistical analysis has been performed appropriately and rigorously.
6. The study focused on fish communities along the waterfront based on relative catch and biomass have changed towards an increased dominance of native warmwater species, preferring vegetation and cover as well as an increase in piscivore presence.
7. This study will be beneficial to biologists, policy makers and other stakeholders in understanding key relationships of species and associated traits that are related to biodiversity and fish community changes. This will also help in restoration of the home of native fish species and provide them suitable ecosystem.

**Feedback/ Suggestions:**

1. The manuscript is well structured and data analysis is done with proper data collection.
2. In results (Line no. 248, 249, 279-287, 291), uniformity may be maintained in writing time periods such as T1-T2, T1-T3, T2-T3 instead writing T2-T1, T3-T1, T3-T2 etc.
3. In results (Line no. 283), biomass declined over time (T2-T1 p < n.s. should be corrected with T1-T2 p = n.s.
4. In Discussion (Line no. - 377), increase in herbivores (+7% median biomass contribution, Fig 8). But, Fig. 8 depicts +9% median biomass contributions of herbivores. Similarly (Line no. - 402), a decrease in planktivores (-5% median biomass contribution, Fig 8). But, Fig. 8 depicts -14% median biomass contributions of planktivores. Just check it and make necessary corrections if required.
5. In Discussion (Line no. 429-233), Sentence “Wide variation in Alewife cohorts …………… in nutrient input as well as anthropogenic development” may be rewritten with proper clause and syntax for better understandings of authors’ view. It may be rephrased into two sentences.
6. In Discussion (Line no. 467-470), Sentence “Embayments field a broader mix of warmwater and coolwater species ………. exposed to upwellings?” may be rewritten with proper clause and syntax for better understandings of authors’ view. It may be rephrased into two sentences.
7. In references, line no. 613, name of a co-author should be corrected as  Lapointe NW;
8. In the same reference (line no. 614), *Cyprinus carpio* should be written in italics.
9. There is need to discuss the limitations of the study in more detail, such as the potential bias of electrofishing and the lack of data on other factors that could be driving changes in the fish community.
10. The authors discuss the potential implications of their findings for future management and restoration efforts. For example, they could discuss which species or ecotypes may be most vulnerable to future changes and how these species or ecotypes could be prioritized in restoration efforts.
11. The study does not account for all of the potential factors that could be driving changes in the fish community, such as climate change and pollution. It is important to keep these other factors in mind when interpreting the results of the study.

At last, I request the authors to kindly make the necessary corrections before final submission of the manuscript.

**Reviewer Recommendation:**

1. I recommend the manuscript for publication in PLOS ONE, with minor revisions.
2. I appreciate the authors' efforts to account for different ecotypes and to identify the key species driving changes in the community.
3. The manuscript presents a novel approach to fish habitat restoration and aquatic biodiversity conservation that is more precise, efficient, and humane.
4. The paper is well-written and easy to follow. The study is clear and convincing.
5. Overall, the manuscript is well-written and informative. I recommend that the authors may revise the manuscript to address the comments and to include the additional suggestions that I have made.
